# Perfusion in the Tissue Surrounding Pancreatic Cancer and the Patient's Prognosis

**DOI:** 10.1155/2014/648021

**Published:** 2014-09-11

**Authors:** Yoshihiro Nishikawa, Yoshihisa Tsuji, Hiroyoshi Isoda, Yuzo Kodama, Tsutomu Chiba

**Affiliations:** ^1^Department of Gastroenterology and Hepatology, Kyoto University Graduate School of Medicine, 54 Kawara-cho, Shogoin, Sakyo-ku, Kyoto 606-8507, Japan; ^2^Department of Radiology, Kyoto University Graduate School of Medicine, 54 Kawara-cho, Shogoin, Sakyo-ku, Kyoto 606-8507, Japan

## Abstract

*Objective*. The objective was to investigate the relationship between prognosis in case of pancreatic cancer and perfusion in tissue surrounding pancreatic cancer using perfusion CT. *Methods*. We enrolled 17 patients diagnosed with inoperable pancreatic adenocarcinoma. All patients were examined by perfusion CT and then underwent chemotherapy using gemcitabine. The time density curve (TDC) of each CT pixel was analyzed to calculate area under the curve (AUC) and blood flow (BF) using a mathematical algorithm based on the single-compartment model. To measure the AUC and BF of tumor (AUC_T_ and BF_T_) and peritumoral tissue (AUC_PTT_ and BF_PTT_), regions of interest were manually placed on the cancer and in pancreatic tissue within 10 mm of proximal pancreatic parenchyma. Survival days from the date of perfusion CT were recorded. Correlation between AUC or BF and survival days was assessed. *Results*. We found a significant correlation between AUC_PTT_ or BF_PTT_ and survival days (*P* = 0.04 or 0.0005). Higher AUC_PTT_ or BF_PTT_ values were associated with shorter survival. We found no significant correlation between AUC_T_ or BF_T_ and survival. *Conclusions*. Our results suggest that assessments of perfusion in pancreatic tissue within 10 mm of proximal pancreatic parenchyma may be useful in predicting prognosis.

## 1. Introduction

Pancreatic ductal adenocarcinoma (PDA) is the fourth leading cause of cancer-related death in the United States [1]. PDA is nearly universally lethal, with 5-year survival rates of less than 5% [1–3]. This poor prognosis is related to early diagnostic difficulties; the disease in more than 80% of patients at the diagnostic stage is already metastatic or locally advanced [2]. Inoperable patients typically undergo gemcitabine-based chemotherapies but with limited effectiveness [4].

Desmoplastic stroma is a histopathological characteristic of PDA [5]. The lack of adequate vasculature due to the presence of desmoplastic stroma is believed to be among the factors leading to resistance to conventional chemotherapies. The low density of vasculature causes poor perfusion, limiting the transport of the anticancer drug from vessel to tissue [6]. Tumor-associated stroma has been reported to increase chemoresistance in PDA [7]. Stromal accumulation of hyaluronan in a mouse model of PDA impaired both vascular function and drug delivery [8]. Accumulating evidence suggests the importance of tumor-associated stroma and vasculature in PDA.

As reported in previous studies, patients with pancreatic cancer may have a history of chronic pancreatitis [9]. Additionally, patients with PDA often have cancer-related pancreatitis [5]. The microstructure of the pancreas in PDA patients tends to be highly desmoplastic, resulting in reduced tissue perfusion. However, recent reports based on mouse PDA model indicate increased perfusion in the tissue surrounding PDA [10]. In human, Radu et al. report that cancer surrounding vasculature was changed due to development of cancer [11]. These studies suggest that perfusion in the tissue surrounding cancer sites may be related to cancer activity. This possibility suggests a need to investigate the relationship between prognosis and perfusion in the tissue surrounding cancer. However, tissue vasculature can be ascertained only through intensive examination (e.g., of pathological specimens), a process that presents major difficulties. For these reasons, how or whether perfusion in the tissue surrounding a cancer relates to cancer activity remains poorly understood.

Recent reports indicate perfusion CT can be used to evaluate tissue vasculature, thereby allowing noninvasive perfusion measurements. Perfusion CT is a type of dynamic CT capable of measuring tissue perfusion based on analyses of time-density curve (TDC) derived from a bolus injection of contrast material. Perfusion CT is reported to be able to obtain nonmorphological information and is valuable for diagnosis in some organs [12]. In the study described here, we applied perfusion CT to investigate the relationship between patient prognosis and perfusion in the tissue surrounding a pancreatic cancer using perfusion CT.

## 2. Materials and Methods

### 2.1. Patients

Between December 2008 and February 2011, our pilot study enrolled 17 patients with inoperable pancreatic adenocarcinoma (PDA). We obtained written informed consent from all patients, and the research protocol was approved by the corresponding institutional review boards. Patients with histologically diagnosed pancreatic adenocarcinoma judged to be inoperable metastatic or locally advanced cancer were enrolled in this study. Diagnoses of locally advanced cancer and/or metastasis were made by a single board-certificated radiologist based on CT and/or MRI findings. All patients were treated using gemcitabine. Patients demonstrating intolerance for the contrast material for dynamic CT were excluded from the study. Our medical chart recorded age, gender, survival days from the date on which perfusion CT was performed, TNM [13], and stage of cancer [14].

### 2.2. Perfusion CT Protocol and Analysis

All patients were examined by perfusion CT and then underwent chemotherapy using gemcitabine. We used multidetector CT (Aquilion 64, Toshiba Medical Systems, Tochigi, Japan) to perform pancreatic perfusion CT [15]. The scanning tube voltage and current were 80 kVp and 40 mA, respectively, resulting in radiation exposures of 60–100 mGy (CTDIvol) [16]. For initial localization of the tumor, a CT study of the abdomen was obtained without contrast material enhancement during a breath hold at the end of expiration; then the CT perfusion examination of the selected area was performed in a single breath hold at end expiration. A supervising radiologist identified the tumor and then placed the predefined scan volume in the* z*-axis to cover the lesion for the CT perfusion study. We referred to other image data sets (e.g., US and MRI) for patients for whom such data sets existed to help identify cancer sites. To reduce respiratory artifacts, a belt over the abdomen was used and patients were instructed to breathe gently during the scan acquisition.

**Figure 1:**
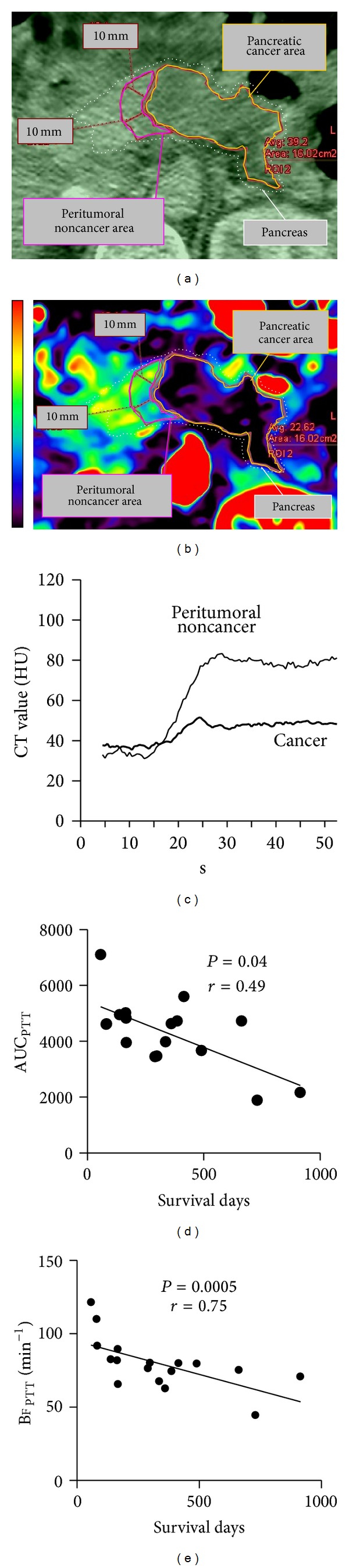
Analysis of pancreatic perfusion image. We analyzed the CT image dataset (of which Panel (a) is an excerpt) to obtain Panel (b). Panels (a) and (b) are magnified by the same factor. Panel (b) is a perfusion image of pancreatic blood flow (BF). Pancreatic BF is indicated by the scale on the left. Colors shift from black to red with increasing BF. The pancreatic regions of interest (ROIs) in Panels (a) and (b) have the same size and location. Panel (c) shows time-density curves for peritumoral noncancer (PTT) and cancer (T) sites for Panels (a) and (b). Panels (d) and (e) show the relationship between pancreatic AUC_PTT_ or BF_PTT_ and survival days, respectively.

Stationary CT scans of four slices were acquired every 0.5 seconds over a period of 54 seconds following intravenous bolus injections of 40 mL of contrast material (Iomeprol 350 mg/mL (molecular weight, 777 kDa)) at 4 mL/second. Perfusion CT scan began 3 seconds after the start of injection. We injected iodinate contrast material through a 20-gauge intravenous cannula, followed by injection of 50 mL of saline solution, in a right cubital vein. The TDC of each CT pixel was analyzed to calculate the area under the curve (AUC) and blood flow (BF) using a mathematical algorithm based on a single-compartment model [17, 18] on workstation (ziostation2, Ziosoft, Tokyo, Japan) (Figures 1(a)–1(c)) [19].

After all of the images were loaded on a dedicated work station, the tumor was defined. TDC of the arterial input was measured by placing a circular region of interest (ROI) within the aorta on a selected image. The arterial TDC was derived automatically by the software. The AUC and BF of tumor (AUC_T_ and BF_T_) and peritumoral tissue (AUC_PTT_ and BF_PTT_) were obtained within a freehand ROI drawn both over the tumor itself and over pancreatic tissue within 10 mm of the juxtaposed proximal pancreatic parenchyma. We drew the largest possible single ROI that could be drawn around each tumor and peripancreatic tissue while still excluding necrosis, calcifications, and cystic or any hemorrhagic areas. The perfusion values were obtained from the parametric maps generated with the software package. Image analysis was performed in consensus by single radiologist (with 11-year experience in abdominal perfusion CT).

### 2.3. Statistical Analysis

We recorded survival days from the date of perfusion CT by chart review and assessed the correlation between AUC or BF and survival days by Spearman's rank correlation test. Data is presented as median (range); *P* values of less than 0.05 were deemed significant. The software used for statistical analysis was JMP (version 9.01, SAS Institute, NC).

## 3. Results

### 3.1. Patients

Between December 2008 and February 2011, our pilot study enrolled 17 patients with inoperable pancreatic adenocarcinoma (PDA). Of these patients, 12 (70.6%) were male and 5 (29.4%) were female. The median age was 63 (36–78). Median survival days from the date on which perfusion CT was performed were 298 days (57–914) (Table 1). According to TNM classification, patients with T4 (tumor involves celiac axis or superior mesenteric artery) and T3 (tumor extends beyond pancreas but no celiac or superior mesenteric artery involvement) [13] numbered 14 (82.4%) and 3 (17.6%), respectively. According to the Japanese classification, 8 patients were stage IVa (locally advanced cancer) and 9 patients were stage IVb (metastatic cancer) [14].All patients were treated with gemcitabine.

### 3.2. Perfusion Data and Survival Days

**Figure 2:**
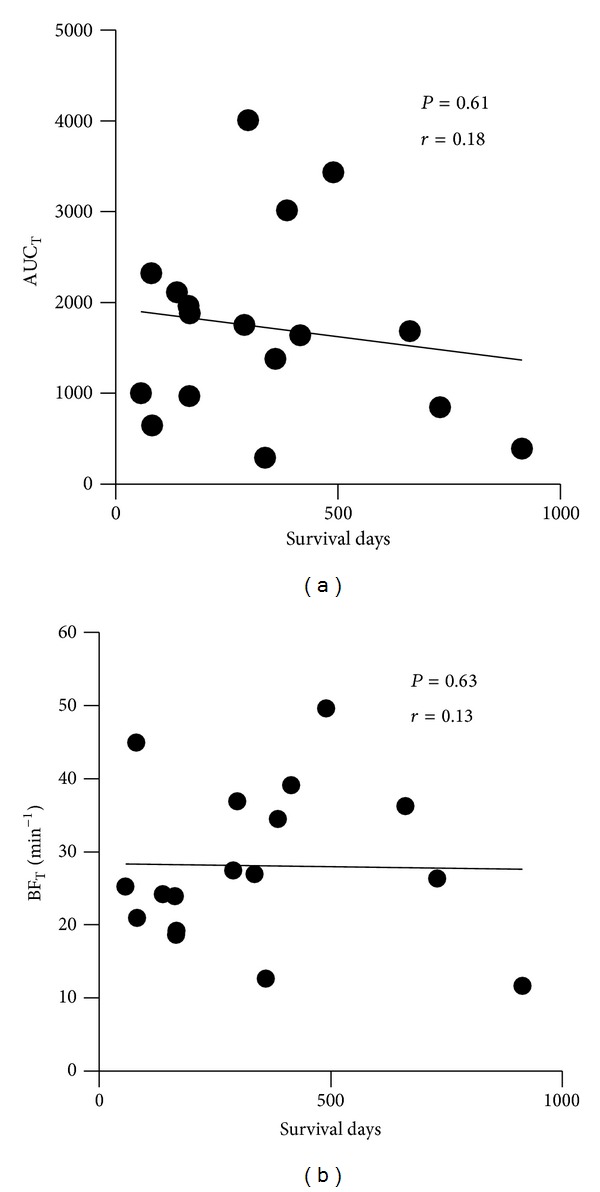
Relationship between AUC_T_ or BF_T_ and survival days. Panels (a) and (b) show the relationship between survival days and pancreatic AUC_T_ or BF_T_, respectively.

We investigated area size, BF, and AUC of TDC in tumors and peritumoral tissue (Table 2). Area size was measured using the ROI on a workstation. We also used this ROI to measure BF and AUC. The area size of pancreatic tumor area and peritumoral area (average ± SD), respectively, were 17.7 ± 24.1 (cm^2^) and 1.9 ± 1.1 (cm^2^). BF_PTT_, AUC_PTT_, BF_T_, and AUC_T_, respectively, were 79.6 ± 17.5 (min^−1^), 2904 ± 726, 28.1 ± 10.7 (min^−1^), and 2353 ± 701. We observed significant correlation between AUC_PTT_ or BF_PTT_ and survival days from the date on which perfusion CT was performed (*P* = 0.04 or 0.0005). Higher AUC_PTT_ or BF_PTT_ values were associated with shorter survival (Figures 1(d) and 1(e)). We found no significant correlation between BF_T_ or AUC_T_ and survival (Figures 2(a) and 2(b)).

## 4. Discussion

In this study, we investigated the relationship between patient prognosis and perfusion in pancreatic cancer and tissue surrounding cancer using perfusion CT. In startling finding, survival days correlated significantly with peritumoral blood flow but not with tumor blood flow.

The results suggest that prognosis is related to increased perfusion in tissue surrounding cancer. Using MR perfusion technique in animal model, Olive et al. have shown that blood flow of peripheral tissue of pancreatic cancer increased [10]. Radu et al. have reported that follicle-stimulating hormone receptor (FSHR) was selectively expressed on the surface of peritumoral vessels [11]; in their report, the authors speculate that FSHR expression may induce VEGF and VEGF receptor 2 signaling in tumor endothelial cells and thereby promote increased vascularization. Pancreatic cancer may alter peritumoral microstructures before invading normal tissue. Thus, increased peritumoral perfusion may be related to cancer activity, as we showed.

As mentioned above, higher perfusion suggests the lower presence of stroma. Reports indicate that poor tumor perfusion is among the factors leading to PDA chemoresistance [4, 10]. As previous study using perfusion MRI reported pathologically [6], the presence of a prominent stromal matrix reduces blood vessel density in PDA tissue. A previous study [10] showed that depletion of tumor associated stromal matrix, using the inhibitor of hedgehog signaling pathway through effect on Smo, increased vasculature and concentration of drug in the tumor tissue and approved prognosis. Beatty et al. also showed that depleting the tumor stroma via activated macrophages using an agonist CD40 antibody improved prognosis in a genetically engineered mouse model of PDA [20]. However, our present study found tumor blood flow unrelated to prognosis. Our evaluation accounted for only one perfusion parameter: tumor blood flow. In fact, there are several perfusion parameters, including tissue blood flow, blood volume, and permeability [21]. Park et al. report that decreased tumor permeability measured by perfusion CT is related to chemosensitivity [22]. Thus, our study leaves open the possibility that another tumoral perfusion parameter may be related to prognosis.

Our investigation presents the following potential limitations. First, while we used the patient survival days as an index of prognosis, prognosis is not necessarily equivalent to chemosensitivity; we did not assess the relationship between perfusion in the tissue surrounding cancer and response rate to gemcitabine. Second, we defined the tissue surrounding cancer as pancreatic tissue within 10 mm of the juxtaposed proximal pancreatic parenchyma. While we assumed this tissue was composed of normal pancreatic tissue, it is certainly possible that it contained a marginal zone of cancerous tissue. Third, we used the software developed by Ziosoft, but differences of perfusion parameters between software programs or their upgrades have been reported, recently [23]. Therefore, our results could change by analyzing other software. Lastly, our study was a pilot study enrolling a limited number of patients.

## 5. Conclusion

Patient prognosis may be related to perfusion in tissue surrounding pancreatic cancer observed with perfusion CT.

## Figures and Tables

**Table 1:** Background information for patients enrolled.

Number	Sex (M/F)	Age (age)	Survival days from date PCT performed	TNM	Stage
1	M	78	57	T4N2M1	IVb
2	M	61	298	T4N0M0	IVa
3	M	59	360	T4N3M1	IVb
4	F	78	80	T3N1M1	IVb
5	M	36	167	T3N0M1	IVb
6	F	63	138	T4N2M0	IVb
7	F	65	662	T4N1M0	IVa
8	M	61	82	T4N0M1	IVb
9	M	49	290	T4N0M0	IVa
10	M	55	166	T4N0M1	IVb
11	F	67	386	T4N0M0	IVa
12	M	64	914	T4N1M0	IVa
13	M	66	415	T4N0M0	IVa
14	M	65	730	T4N0M1	IVb
15	M	46	164	T4N0M0	IVa
16	F	66	490	T3N0M1	IVb
17	M	46	336	T4N0M0	IVa

	12/5	63.0 ± 11.1	298 ± 246		

Figures (median ± SD) for age and survival days from date perfusion CT (PCT) performed appear at the bottom of each column.

**Table 2:** Area, blood flow, and area under curve with injection of contrast material in pancreatic tumor and peritumoral tissue.

Number	Area of pancreatic tumor (cm^2^)	Area of peritumoral tissue (cm^2^)	BF_PTT_ (min^−1^)	AUC_PTT_	BF_T_ (min^−1^)	AUC_T_
1	28.8	25.2	121.4	3511	999	1618
2	98.6	36.9	80.15	2946	4004	3337
3	7.4	12.63	62.65	2173	1376	2529
4	3.8	44.9	109.9	3706	2321	2689
5	4.0	19.15	65.6	1786	1879	2604
6	27.8	24.16	82.48	2593	2108	2256
7	3.0	36.22	75.2	3277	1682	2344
8	22.2	20.9	91.7	3791	645	3791
9	2.9	27.4	76.4	2800	1750	2988
10	19.1	18.6	89.52	3340	966	1204
11	1.6	34.47	74.35	3527	3012	2622
12	7.5	11.6	70.8	2778	386	1796
13	2.7	39.1	79.9	2763	1638	2779
14	3.7	26.3	44.4	1413	844	1258
15	16.0	23.9	81.78	3312	1959	1909
16	44.4	49.6	79.5	1931	3430	1741
17	7.3	26.9	67.5	3717	289	2537

	17.7 ± 24.1	1.9 ± 1.1	79.6 ± 17.5	2904 ± 726	28.1 ± 10.7	2353 ± 701

BF_PTT_ and BF_T_, respectively, represent blood flow (BF) of peritumoral tissue (PTT) and pancreatic tumor (T) as determined by perfusion CT. AUC_PTT_ and AUC_T_, respectively, represent area under curve (AUC) with bolus injection of contrast media for PTT and T. Measurement results (average ± SD) appear at bottom of each column.
